# DNA-free high-quality RNA extraction from 39 difficult-to-extract plant species (representing seasonal tissues and tissue types) of 32 families, and its validation for downstream molecular applications

**DOI:** 10.1186/s13007-023-01063-5

**Published:** 2023-08-11

**Authors:** Shina Sasi, Saranya Krishnan, Preshobha Kodackattumannil, Aysha AL Shamisi, Maitha Aldarmaki, Geetha Lekshmi, Martin Kottackal, Khaled M. A. Amiri

**Affiliations:** 1 Khalifa Center for Genetic Engineering and Biotechnology, United Arab Emirates University, P.O. Box 15551, Al Ain, United Arab Emirates; 2 Department of Biology, College of Science, United Arab Emirates University, P.O. Box 15551, Al Ain, United Arab Emirates

**Keywords:** CTAB, *Conocarpus erectus*, Date palm, *Prosopis cineraria*, RNA extraction, TRIzol

## Abstract

**Background:**

High-purity RNA serves as the basic requirement for downstream molecular analysis of plant species, especially the differential expression of genes to various biotic and abiotic stimuli. But, the extraction of high-quality RNA is usually difficult from plants rich in polysaccharides and polyphenols, and their presence usually interferes with the downstream applications. The aim of the study is to optimize the extraction of high-quality RNA from diverse plant species/tissues useful for downstream molecular applications.

**Results:**

Extraction of RNA using commercially available RNA extraction kits and routine hexadecyltrimethylammonium bromide (CTAB) methods did not yield good quality DNA-free RNA from *Prosopis cineraria*, *Conocarpus erectus*, and *Phoenix dactylifera*. A reliable protocol for the extraction of high-quality RNA from mature leaves of these difficult-to-extract trees was optimized after screening nine different methods. The DNase I-, and proteinase K treatment-free modified method, consisting of extraction with CTAB method followed by TRIzol, yielded high-quality DNA-free RNA with an A_260_/A_280_ and A_260_/A_230_ ratios > 2.0. Extraction of RNA from *Conocarpus*, the most difficult one, was successful by avoiding the heat incubation of ground tissue in a buffer at 65 ^o^C. Pre-warming of the buffer for 5–10 min was sufficient to extract good-quality RNA. RNA integrity number of the extracted RNA samples ranged between 7 and 9.1, and the gel electrophoresis displayed intact bands of 28S and 18S RNA. A cDNA library constructed from the RNA of *P. cineraria* was used for the downstream applications. Real-time qPCR analysis using the cDNA from *P. cineraria* RNA confirmed the quality. The extraction of good quality RNA from samples of the desert-growing *P. cineraria* (> 20-years-old) collected in alternate months of the year 2021 (January to December covering winter, spring, autumn, and the very dry and hot summer) proved the efficacy of the protocol. The protocol’s broad applicability was further validated by extracting good-quality RNA from 36 difficult-to-extract plant species, including tissues such as roots, flowers, floral organs, fruits, and seeds.

**Conclusions:**

The modified DNase I and Proteinase K treatment-free protocol enables to extract DNA-free, high-quality, intact RNA from a total of 39 difficult-to-extract plant species belonging to 32 angiosperm families is useful to extract good-quality RNA from dicots and monocots irrespective of tissue types and growing seasons.

**Supplementary Information:**

The online version contains supplementary material available at 10.1186/s13007-023-01063-5.

## Background

High-quality DNA-free RNA is mandatory for a number of plant molecular biology applications such as reverse transcription polymerase chain reaction (RT-PCR), real-time fluorescent quantitation polymerase chain reaction (qPCR), cDNA library construction, northern blot, RNase protection assay, in situ hybridization, subtractive hybridization, and gene expression analysis. Also, good-quality RNA is the prime requirement for RNA-seq analysis which is highly used for gene expression analysis in different tissues and plants under stress, detection of mutations, alternative splicing, and post-transcriptional modifications.

Plants are very diverse in their biochemical profile, and the extraction of RNA at a quality desired for downstream applications is difficult with plants abundant in polysaccharides, proteins, and secondary metabolites [1, 2]. Polysaccharides co-precipitate with RNA in low ionic strength buffers, which constitute potent enzyme inhibitors that reduce yield and result in poor-quality RNA by significantly affecting the extraction procedure [1, 2]. Polyphenols bind irreversibly to RNA and proteins to form high-molecular-weight complexes [1, 2]. Both adversely affect downstream operations like gene expression [3] and RNA-seq analysis [4].

Plant growth stage (immature and mature), physiological status of the samples, age of tissue (i.e., young freshly expanding vs. mature fully expanded but non-senescing tissue), type of tissue (leaf, root, stem, flower, fruit tissues and seeds), growing season especially under abiotic conditions, biochemical profile of the plants, type of plants (herbs, shrubs, trees) and conditions of the material (dry, rigid fibrous material) are the factors affecting the extraction of high-quality RNA [1, 5, 6]. Immature tissues, preferably the seedling leaves, are mostly selected to extract high-quality RNA as they are low in polysaccharides and polyphenols [4, 5, 7, 8]. Extraction of high-quality RNA from plants growing in different environments such as deserts, mountains, seawater, or experimental stress (abiotic and biotic) conditions seems complicated due to high levels of inhibitory/RNA binding metabolites [5, 9, 10]. In the case of plants, trees, in particular having short periods of the growing season, i.e., the emergence of new foliage, mature leaves, or tissues, are the sources for extraction of RNA for downstream applications. The extraction of RNA, even from different species of the same genus, requires optimization for the protocol to yield high-quality, intact RNA. Various methods have been developed for RNA extraction from different plant tissues [5, 6, 11, 12]. RNA extraction protocols across planta use guanidinium thiocyanate, CTAB, or sodium dodecyl sulfate (SDS) based methods [5, 6, 13, 14]. However, the methods must be optimized for specific species and even for the nature of the target tissue [4]. Successful extraction of RNA using CTAB, TRIzol, and TRIzol Plus RNA Purification kits has been reported [4–7, 12, 15]. The kit formats are highly useful for extracting RNA from the model plants [16]. CTAB-based extraction and its modifications have been reported as successful for high-quality RNA from recalcitrant plants [4–6]. Further, special precautions are required for RNA isolation as it has a very short half-life once extracted from cells or tissues and is susceptible to degradation [13]. Our attempt to extract a fair amount of good quality DNA-free RNA for downstream applications of *Prosopis cineraria, Conocarpus erectus, Phoenix dactylifera* was unsuccessful using different established protocols.

*Prosopis cineraria* (L.) Druce, a member of the legume family (Fabaceae), is one of the prime choices for desert afforestation programs due to their high adaptability to thrive in extreme environmental conditions of deserts (https://sdgs.un.org/partnerships/give-ghaf-tree-planting-program#:~:text=With%20over%2075%2C000%20seeds%20planted,air%2 C%20for%20generations%20to%20come). *Prosopis* spp. are rich in tannins and polysaccharides [17], and these compounds are considered problematic to yield good quality RNA suitable for downstream applications. Total RNA extraction from seedling tissues of *Prosopis* spp. has been documented by CTAB methods [18, 19] and commercial kits [20].

*Conocarpus erectus* L., commonly known as button mangrove, belongs to the Combretaceae is an associate mangrove shrub growing on shorelines in tropical and subtropical regions around the world. The plant is a rich source of polyphenols and is reported to contain more than 12 phenolic compounds, mainly flavonoids and tannins [21, 22]. There is no report of total RNA extraction from this polysaccharide and phenolic-rich plant species to date.

*Phoenix dactylifera* L. (Arecaceae), the Date palm, is a dioecious perennial monocot with several varieties and is the most important fruit-bearing crop in arid regions of the Middle East [23, 24]. Palms are rich in polysaccharides and polyphenolics and have leaves with a waxy cuticle and high fiber content [25], making extracting total RNA from the leaves very difficult. The date palm is reported to contain phenolic and their positional isomers, which usually interfere with the successful isolation of nucleic acids [26]. Total RNA extraction was achieved mainly from seedling tissues of Date palm [27–29].

To extract quality RNA from these plants for our downstream applications, we have tried different RNA extraction protocols such as Qiagen’s RNeasy Plant Mini kit, PureLink RNA Mini kit (Invitrogen, USA), Spectrum Plant Total RNA kit (Sigma, USA), Plant/Fungi DNA/RNA isolation kit (Norgen Biotek, USA), TRIzol (Ambion, USA); TRI Reagent (Sigma, USA), CTAB-based [30], and hybrid methods. We have developed a modified protocol suitable for extracting high-quality, intact RNA without DNase and proteinase K treatment, which was validated by cDNA synthesis followed by gene cloning, cDNA library preparation, and RT-qPCR analysis. Further, the applicability of the optimized RNA extraction protocol was evaluated by extracting high-quality RNA from 36 other difficult-to-extract non-model plant species consisting of different tissues such as seed, fruit (mature and immature), male and female flowers, floral parts (anther, pollen) and roots and tissues with respect to the season.

## Materials and methods

### Plant samples

Mature leaves of *Prosopis cineraria* (Fabaceae), *Conocarpus erectus* (Combretaceae), and *Phoenix dactylifera cv*. Fard (Arecaceae) were used to optimize a high-quality RNA extraction protocol. The leaf samples of *P. cineraria* were collected in August (42–47 ^o^C temp.) 2020 from > 20-year-old trees growing in the desert of Sweihan area (24°17’18.3"N 55°43’36.2"E), Al Ain, UAE. The tissue samples for the extraction of the RNA with respect to the season from *P. cineraria* were collected in alternate months (January to December) of the year 2021. The leaf samples of *C. erectus* and Date palm were collected from the plants (> 20-years-old) growing nearby our Center. The plant tissues collected were frozen in liquid-Nitrogen (LN_2_) and stored at ^−^80 °C to extract RNA for downstream studies.

#### Sample tissue preparation

Stringent measures were taken to eliminate the RNase activity from the beginning of the extraction procedure until the end and thereafter in the handling of the RNA. RNase*Zap*® RNase Decontamination Solution (Cat. no. AM9780; Ambion, USA) was used to eliminate the RNase activity during the extraction of the RNA. In all protocols, the tissues were ground to a fine powder in a pre-chilled mortar and pestle using liquid nitrogen and used immediately or stored at ^−^80 °C for later use.

#### Kit protocols

The extraction of total RNA using RNeasy Plant Mini Kit (Cat. no. 69106; Qiagen, Germany), PureLink RNA Mini kit (Cat. no. 12183,018 A; Invitrogen, USA), Spectrum Plant Total RNA kit (Cat. no. STRN50; Sigma, USA), and Plant/Fungi DNA/RNA isolation kit (Cat. no. 26200; Norgen Biotek, USA,) was performed as per the manufacturers’ instructions. An amount of 25–50 mg of finely powdered tissue was used to extract total RNA for the kit protocols.

### Trizol Protocol

In the case of TRIzol (Cat. no. 15596018; Ambion, USA) and TRI Reagent (Cat. no. T9424; Sigma, USA), ~ 50–100 mg/ml finely powdered tissue was transferred for the extraction and carried out as follows.

1. Transferred 50–100 mg powdered, frozen tissue into 1 ml TRIzol/Sigma TRI Reagent in a 2 ml microcentrifuge tube. Mixed well by vigorous shaking and incubated for 5 min at room temperature (RT, 22–23 °C).
2. Centrifuged the tubes at 14,000 rpm for 10 min at RT.
3. The aqueous phase was transferred into a 1.5 ml microcentrifuge tube and added 0.2 ml chloroform. Mixed well by vigorous shaking, incubated for 5 min at RT, and centrifuged for 10 min at 14,000 rpm.
4. Transferred the aqueous phase into a 1.5 ml microcentrifuge tube and added 0.2 ml absolute ethanol (Added 1 vol. of isopropanol for Sigma TRI Reagent). Mixed well by inversion.
5. Decanted the supernatant after centrifuging at 15,000 rpm for 20 min. The pellet was washed with 500 µl of 70% (v/v) ethanol for 3–5 min. Carefully decanted or pipetted out the supernatant and repeated the step.
6. The air-dried pellet was dissolved in 50 µl of RNase-free water.

### TRIzol-column hybrid protocol

In this method, TRIzol (Ambion) was used as it was found superior to Sigma TRI Reagent.

Followed the TRIzol protocol up to step 4.

5. Transferred the solution onto an RNeasy (pink) column of the Qiagen kit (or the column of PureLink RNA isolation kit/Spectrum RNA isolation kit).
6. Centrifuged 1 min at 14,000 rpm and discarded the flow-through.
7. Added 500 µl RPE buffer (Qiagen). Centrifuged 30–60 s at 12,000 rpm. Discarded the flow-through. Repeated the step.
8. Centrifuged the column at 12,000 rpm for 1 min.
9. Transferred the column to a new RNase-free 1.5 ml microcentrifuge tube. Added 50 µl of RNase-free water into the center of the column, incubated for 3–5 min at RT, and centrifuged for 1 min at 12,000 rpm.

### CTAB protocol

The hexadecyltrimethylammonium bromide (CTAB; Cat. no. 52365; Sigma, USA) protocol of Chang et al. [30] was tried to extract RNA. The extraction using CTAB buffer was followed by SSTE buffer [30]. All the solutions were prepared with nuclease-free water. The composition of the CTAB extraction buffer was: 2% (w/v) CTAB, 2% (w/v) polyvinylpyrrolidone K-40 (PVP, Cat. no. PV40; Sigma, USA), 100 mM Tris-HCl (pH 8.0; Trizma base, Cat. no. 1503; Sigma, USA), 25 mM ethylenediaminetetraacetic acid disodium salt dihydrate (EDTA; Cat. no. EA023, Sigma, USA), 2.0 M sodium chloride (NaCl; Cat. no. 1103414; Sigma, USA), 0.5 g/1 spermidine (free acid, S2626-1G, Sigma, USA), 5% (v/v) β-mercaptoethanol (added just before use, Cat. no. 63689; Sigma, USA). SSTE buffer consisted of 1.0 M NaCl, 0.5% (w/v) sodium dodecyl sulfate (SDS, Cat. no. L3771; Sigma, USA), and 10 mM Tris-HC1 (pH 8.0).

### SDS-LiCl method

The protocol reported as universal by Vennapusa et al. [5] was attempted to extract the RNA precisely as described.

### Modified protocol of the present study

The optimized protocol of the present study was a modification of Chang et al. [30]. It consisted of an extraction using the CTAB buffer [30] and was followed by TRIzol (Ambion), which is described below step by step.

1. Prepared 5 ml CTAB extraction buffer by adding 5% (v/v) β-ME in a 15 ml Falcon tube and incubated at 65 °C for 5–10 min in a pre-heated heat block.
2. Transferred the fine tissue powder (200–500 mg of ground tissue) into 5 ml extraction buffer, mixed well by vortexing (30 s), and shook thoroughly. Incubated for at least 5 min at RT on a Nutating mixer (or similar kind) and centrifuged at 12,000 rpm for 10 min.
3. Transferred the aqueous phase to a new 15 ml tube and added an equal volume of CIA (24:1). Mixed by shaking the tube thoroughly, incubated at least 5 min at RT on a Nutating mixer and centrifuge at 12,000 rpm for 10 min.
4. Repeated step 3.
5. Transferred the aqueous phase to a new tube and added 1/4 vol. (for 4 ml, added 1 ml) 10 M LiCl, mixed by inversion, and precipitated overnight at 4 ^o^C (or 4–6 h at ^-^20 ^o^C).
6. Centrifuged at 15,000 rpm (> 20,000 x*g*) for 20–30 min at 4 ^o^C, decanted and drained off all the supernatant by keeping the tube upside down on a paper towel or pipetted out.
7. Dissolved the pellet in 1 ml TRIzol (Ambion), vortexed, and shook vigorously. Incubated for 3–5 min at RT.
8. Centrifuged the tubes at 15,000 rpm for 10 min and transferred the aqueous phase into a 1.5 ml microcentrifuge tube.
9. Added 0.2 ml of chloroform, mixed well by vortexing and vigorous shaking. Incubated for 3–5 min at RT and centrifuged for 10 min at 15,000 rpm (> 20,000 x*g*).
10. Transferred the aqueous phase into a 1.5 ml microcentrifuge tube and added an equal volume of CIA or chloroform. Mixed well by vigorous shaking, incubated for at least 5 min on a Nutating mixer at RT, and centrifuged for 10 min at 14,000 rpm.

Optional: A repeat of step 10 with CIA was good for the complete elimination of proteins, especially in the case of challenging tissue types such as fruit, tuber, etc.

11. Transferred the aqueous phase into a 1.5 ml microcentrifuge tube and added 0.5 vol. of ethanol (if isopropanol, add 1 vol. of isopropanol). Mixed well by inversion.
12. Centrifuged at 15,000 rpm for 10 min. Carefully decanted or pipetted out the supernatant.
13. Washed the pellet with 500 µl of 70% (v/v) ethanol for 3–5 min. Centrifuged at 15,000 rpm for 1–2 min. Carefully decanted or pipetted out the supernatant. Repeated the step and air-dried the pellet.
14. Added 50 µl of RNase-free water and dissolved the pellet completely.

### RNA quality and quantity assessment

The quantity and purity of the extracted RNA samples were determined by measuring 2 µl in a Nanodrop spectrophotometer (Nanodrop 2000, Thermo Scientific). The quality of the RNA samples was assessed by loading 1 µg total RNA on a Hydragreen (non-toxic nucleic acid stain, ACT gene, NJ, USA) added 1% (w/v) agarose (Cat. no. 8010.00; Conda Lab, Spain) gel, and were documented using GelDoc™ EZ Gel Documentation System (Bio-Rad, USA).

### RNA integrity determination

RNA integrity Number (RIN) values were determined based on the entire electrophoretic RNA sample trace on an Agilent 2100 Bioanalyzer (Expert B.02.10.SI764). One µl of extracted total RNA was loaded to determine the integrity and concentration in the Bioanalyzer with the Plant RNA Pico chip assay following the manufacturer’s instructions (Agilent Technologies, Santa Clara, CA, USA). The Bioanalyzer uses the electrophoretic technology on a chip to separate RNA fragments by size, read by laser-induced fluorescence, is translated into electropherograms (bands and peaks), and displays the distribution and relative amounts of differently sized RNAs. The measuring of the RNA integrity was carried out as reported earlier [4, 31], based on two metrics: the ratio of the large (28S) to small (18S) ribosomal RNA subunits (28S/18S) and the RIN.

**Downstream analysis of the extracted RNA**.

a) cDNA synthesis, amplification of genes using PCR, and gene cloning.

The RNA sample isolated from *P. cineraria* were reverse transcribed using Invitrogen SuperScript III Reverse Transcriptase using oligodT(20) according to the manufacturer’s instructions (Cat. no. 18080093; ThermoFisher Scientific, USA). PCR using the synthesized cDNA with gene-specific primers for *HSF6a* and *HSF7b* (Table 1) was carried out in a 50 µl reaction mixture using the Phusion High-Fidelity Taq polymerase kit (Cat. no. M0530L; NEB, USA) following the manufacturer’s protocol adding 1–2 µL cDNA. The PCR program was as follows: initial denaturation at 98 °C for 30 s followed by identical 30 cycles of 10 s denaturing at 98 °C, 20 s annealing at 55 °C, and 40 s extension and final extension of 10 min at 72 °C. The PCR products were resolved on 1% (w/v) agarose gel and were documented using the GelDoc. The amplified fragments extracted from the gel using NucleoSpin gel and PCR Clean-up (Cat. no. 740609.50 S; Macherey-Nagel, Germany) were cloned in a pCR-Blunt vector (ZeroBlunt PCR cloning kit, Cat. no. K2700-20; Invitrogen, USA) and sequenced (Macrogen, Korea). The sequences were confirmed by BLAST (NCBI) similarity search.

**Table 1:** Sequences of the primers used in the present study

Primer Name	5’- 3’ Sequence
HSF6a-F	ATGAATCGTATCGATGATGGTC
HSF6a-R	TCAATAACCAAGTTCTTCAGACAA
HSF7b-F	ATGAACTATTTGTACCCTGTGAAAGA
HSF7b-R	TCATTTAGGACTTGACCCTAAGTAAC
HSF6a-qF	GTCAGACAGCTCAACACCTATG
HSF6a-qR	ACTGCTCTTCCTCCTCCTTAT
HSF7b-qF	ACCCTACCACTAGCCACATA
HSF7b-qR	GCTTGAAGTATCGAGGGAGAAG
Actin-qF	GAAGCTGCAGGTATCCATGAGACC
Actin-qR	AGGCAGTGATCTCCTTGCTCATC
M13(-20)-F	GTAAAACGACGGCCAG
M13-R	CAGGAAACAGCTATGAC

q – represents q-PCR primers

#### Real-Time-qPCR

Gene expression analysis by synthesizing the first strand cDNA from 1 µg RNA isolated from *P. cineraria* was carried out using QuantiTect Reverse Transcription kit (Cat. no. 205311; Qiagen, Germany) following manufacturer’s instructions in a 20 µl reaction mixture and the cDNA synthesized was stored at ^−^20 ^o^C until use. RT-PCR analyses were carried out in reactions containing 2 µl of diluted (1:4) cDNA, 200 nM primers, and 5 µl of 2X Fast SYBR Green PCR master mix (Cat. no. 4385614; Applied Biosystems, USA) in a 10 µl total volume using a StepOnePlus™ Real-Time PCR system (Applied Biosystems, USA). The *HSF* primers (Table 1) were used for the relative expression of the *HSFs*. The qRT-PCR reactions were performed following the fast-thermal cycles: 50 ^o^C for 2 min, 95 ^o^C for 20 s, followed by 40 cycles of 95 ^o^C for 3 s and 60 ^o^C for 30 s. The actin gene (Table 1) was used as the reference gene. The 2^−∆CT^ method [32] was used to calculate the relative expression level.

#### cDNA library preparation

The cDNA library was prepared using the CloneMiner II cDNA library construction kit (A1180; Invitrogen, USA) as per the manufacturer’s instructions. The cDNA samples following the kit procedure were electroporated into ElectroMAXTM DH10BTM competent cells using Electroporator (Bio-Rad, USA), and the entry clones derived from BP recombination with pDONRTM 222 (Invitrogen, USA) were sequenced using M13(–20)-F and M13-R primers (Table 1).

### Application of the optimized protocol to different plant species and tissues

The applicability of the modified protocol was evaluated by extracting RNA from different plant species and tissues of various angiosperm (monocot and dicot) families, and the details are provided in Additional file 1. The different plant species and tissues collected from various locations stored at -80 ^o^C were powdered into fine powder using LN_2_. RNA was extracted from the powdered tissue using the optimized (CTAB-TRIzol) protocol described.

### Statistics

All the RNA extraction procedures of *P. cineraria, C. erectus* and *P. dactylifera* were repeated five times, and three replicates of other plants/tissues. Data represent the mean ± SE and were evaluated at a 5% level of significance.

## Results and discussion

Successful extraction of high-quality RNA with no degradation and traces of DNA, proteins, polyphenols, and polysaccharides accelerates fundamental and applied research on plant biology. Good quality RNA is essential for diverse downstream molecular studies such as gene expression profiling, subtractive hybridization, construction of cDNA libraries, and RNA-Seq analysis. Optimization of RNA extraction protocol specific to plant species or even to different tissues at high purity is mandatory for trustworthy end results in downstream molecular applications. Polyphenolic components during the extraction of RNA undergo oxidization readily and bind to RNA and co-precipitate, which further cause RNA degradation and become problematic in downstream studies [33].

Lysis of cells, the primary step of RNA extraction, releases large quantities of contaminants that usually hamper RNA extraction and/or result in inhibition in the downstream molecular studies. The RNA extraction buffer plays a crucial role in the rapid denaturation of nucleases and stabilization of RNA to obtain good-quality RNA [4, 5]. Commercially available kits and reagents for the extraction of RNA are simple, rapid, non-toxic, and usually yield high-quality RNA from plant tissues, especially of the model plants: *Arabidopsis*, *Brachypodium*, *Setaria*, and tobacco [11, 34–36]. Nevertheless, the use of these kits or reagents is inefficient in yielding high-quality RNA in sufficient amounts in the case of plants that are rich in polyphenols and polysaccharides [5, 33]. In the present study, the proprietary kits of Qiagen, Invitrogen, and Norgen, and the widely used RNA extraction reagent, TRIzol (Ambion)/TRI Reagent (Sigma), did not yield good quality RNA from the mature leaves of the plant species of the present study (Table 2). In all the cases, the A_260_/A_280_ and A_260_/A_230_ ratios were below 1.8 due to the presence of proteins and polysaccharides. In the present study, though TRIzol (Ambion) was better than TRI Reagent (Sigma), the quality was low (Table 2). The unsuitability of Trizol has also been accomplished in different plant tissues [4, 37]. Trizol reagent, though its success varies with respect to the manufacturers, has been widely used for rapid RNA extraction in several plant species [4, 15, 38]. However, Trizol reagents have proved unsuitable for extracting RNA from plant tissues rich in polysaccharides and polyphenolics [39]. In the TRIzol method, the use of RNA binding columns of commercial kits after ethanol precipitation enabled easy handling and early completion of the protocol, but the amount and quality were low (Table 2). The commercial kits and the TRIzol method did not efficiently separate RNA from proteins and other organic compounds of the plant species of the present study with high levels of polysaccharide and polyphenol (Table 2). This has been pointed out as one of the reasons for the degradation of RNA extracted [40]. In the case of TRIzol, though the quality of the RNA was low, the RNA samples were free of DNA; the centrifugation of the tissue in TRIzol, i.e., before the addition of chloroform, was the reason for the DNA-free status.

**Table 2:** Purity and yield of RNA extracted from leaves of different plants as to different protocols

Protocol	*Prosopis cineraria*			*Conocarpus erectus*			*Phoenix dactylifera*		
	A_260/280_ ratio	A_260/230_ ratio	RNA yield (ng/µl)	A_260/280_ ratio	A_260/230_ ratio	RNA yield (ng/µl)	A_260/280_ ratio	A_260/230_ ratio	RNA yield (ng/µl)
Qiagen	1.54 ± 0.05^c^	1.19 ± 0.03^d^	26.4 ± 1.9^ g^	1.42 ± 0.07^e^	1.13 ± 0.05^e.g.^	19.0 ± 1.9^ g^	1.61 ± 0.07^c^	1.58 ± 0.08^d^	31.9 ± 2.1^ g^
Norgen	1.70 ± 0.08^b^	1.63 ± 0.07^c^	61.9 ± 7.2^f^	1.48 ± 0.09	1.30 ± 0.09^f^	59.5 ± 4.1^f^	1.75 ± 0.04^b^	1.72 ± 0.04^c^	70.1 ± 5.9^f^
Sigma	1.50 ± 0.09^c^	1.29 ± 0.08^d^	27.5 ± 3.2^ g^	1.47 ± 0.06^e^	1.21 ± 0.06^f^	23.5 ± 2.1^ g^	1.60 ± 0.05^c^	1.62 ± 0.04^d^	38.6 ± 3.3^ g^
Invitrogen	1.56 ± 0.07^c^	1.31 ± 0.04^d^	29.9 ± 4.4^ g^	1.51 ± 0.04^d^	1.19 ± 0.06^ fg^	56.7 ± 5.2^f^	1.59 ± 0.09^c^	1.70 ± 0.07^c^	36.8 ± 2.6^ g^
TRI Reagent Sigma	1.70 ± 0.06^b^	1.67 ± 0.05^c^	122.8 ± 6.5^e^	1.54 ± 0.08^ cd^	1.43 ± 0.03^de^	88.8 ± 3.5^e^	1.66 ± 0.07^c^	1.61 ± 0.06^d^	132.1 ± 7.1^d^
TRIzol Ambion	1.74 ± 0.07^b^	1.72 ± 0.07^c^	151.4 ± 5.7^c^	1.66 ± 0.05^c^	1.52 ± 0.06^de^	121.2 ± 7.3^d^	1.75 ± 0.09^c^	1.71 ± 0.05^c^	153.5 ± 5.4^d^
TRIzol + Column (Qiagen)	1.75 ± 0.04^b^	1.71 ± 0.05^c^	133.5 ± 5.1^de^	1.68 ± 0.07^c^	1.59 ± 0.08^c^	56.6 ± 2.9^f^	1.74 ± 0.08^c^	1.72 ± 0.07^c^	69.9 ± 4.4^f^
CTAB	1.98 ± 0.09^a^	2.05 ± 0.05^b^	414.2 ± 17.8^b^	1.91 ± 0.09^b^	1.89 ± 0.08^b^	210.9 ± 21.1^b^	2.00 ± 0.09^a^	2.09 ± 0.07^a^	451.8 ± 26.2^b^
*SDS-LiCl*	1.73 ± 0.05^b^	1.68 ± 0.06^c^	312.1 ± 13.9^c^	1.68 ± 0.06^c^	1.58 ± 0.04^c^	153.5 ± 31.0^c^	1.76 ± 0.06^b^	1.79 ± 0.07^b^	322.0 ± 23.5^c^
CTAB* Modified	2.11 ± 0.09^a^	2.24 ± 0.08^a^	1222.8 ± 32.5^a^	2.17 ± 0.08^a^	2.27 ± 0.07^a^	907.3 ± 25.5^a^	2.12 ± 0.06^a^	2.19 ± 0.05^a^	1008.6 ± 28.5^a^

*Optimized protocol in the present study. Data represent the mean ± SE of 5 replicates. RNA dissolved in 50 µl Nuclease-free water. Values followed by different letters are significantly different at the 5% level. The samples of desert-growing *P. cineraria* were collected in August 2020

Qiagen - RNA using RNeasy Plant Mini Kit (Cat. no. 69106; Qiagen), Invitrogen - PureLink RNA isolation kit, Sigma - Spectrum Plant Total RNA kit (Cat. no. STRN50), and Norgen- Plant/Fungi DNA/RNA isolation kit (Cat. no. 26200) CTAB – [30]; SDS-LiCl – [5]

CTAB-based protocols were widely used to extract RNA from plant tissues, and success has been accomplished in several plant species, especially where the kit formats were unsuccessful [4, 5, 14]. However, the yield and quality varied with respect to the plant tissues [4]. In oil palm, the total RNA extracted from suspension cells and embryogenic callus using the CTAB protocol was useful for subsequent RT-PCR and real-time RT-PCR [41]. But the method was reported as unsuitable for isolating RNA from the leaves of coconut palm [37]. In the present study, the _A260/230_ ratios of the extracted RNA using the modified CTAB method of Chang et al. [30], which was reported as efficient for several recalcitrant plant species was inferior and always displayed DNA contamination (Table 2), which required additional procedures to have DNA-free RNA. Nevertheless, the ratio of the CTAB method was superior to that of the kit formats and TRIzol (Table 2). Several documented results emphasize that CTAB-based protocols as the most efficient technique to extract RNA from forest trees [42] and various species [4]. The protocol of Vennapusa et al. [5], claimed as universal, showed clear DNA bands in their gel images and the RNA extracted with their protocol in the present study. DNase I treatment necessitates additional procedures, which usually result in insufficient RNA for downstream applications [8, 43, 44]. The presence of impurities, such as salts or proteins, can affect the movement of the RNA in agarose gel. During electrophoresis, the RNA extracted from protocols other than the optimized one, RNA loaded wells of the gel upon documentation displayed fluorescence, which is usually due to the presence of impurities (polysaccharides/proteins).

**Fig. 1:**
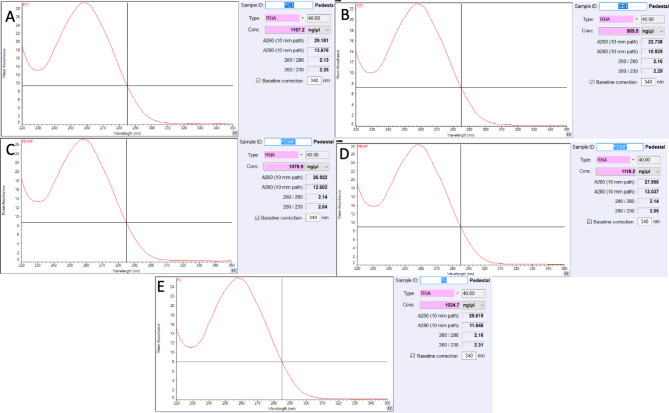
Nanodrop quantitation of the extracted RNA using optimized (CTAB-TRIzol) method. (A) Leaves of *Prosopis cineraria*; (B) Leaves of *Conocarpus erectus*; (C) Immature fruits of *Phoenix dactylifera*; (D) Male flower of *P. dactylifera*; (E) Leaves of *Prosopis juliflora*

**Fig. 2:**
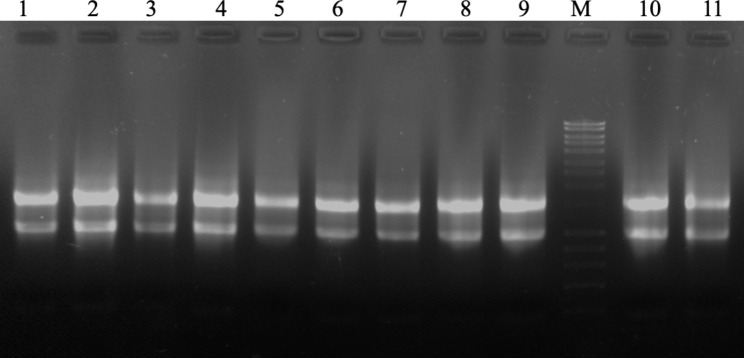
Gel electrophoresis of isolated RNA from different plant species. (1) Mature leaves of *Prosopis cineraria*; (2) Mature leaves of *Phoenix dactylifera*; (3) Mature leaves of *Conocarpus erectus*; (4) Pollen of *P. dactylifera*; (5) Roots of *P. dactylifera*; (6) Male flower of *P. dactylifera*; (7) Mature fruits of *Prunus salicina*; (8) Mature leaves of *Litchi sinensis*; (9) Immature stem of *Leptadenia pyrotechnica;* (10) Mature leaves of *Memecylon umbellatum*; 11. Endosperm of *Cocos nucifera*. (M − 1 kb plus DNA ladder)

CTAB protocol of Chang et al. [30] followed by TRIzol extraction of the present study, facilitated the isolation of high quantities of DNA-free good quality RNA from the selected woody recalcitrant species (Table 2). This combined method improved the quality of the RNA significantly compared to that of the CTAB and TRIzol methods individually (Table 2). The protocol yielded a high amount of DNA-free RNA with A_260_/A_280_ and A_260_/A_230_ ratios > 2.0 (Table 2; Fig. 1A-E). The ratios indicated high purity and the polyphenol and polysaccharide contamination-free status of the RNA samples. In the present study, the extraction of the precipitated RNA of the CTAB method followed by TRIzol efficiently eliminated the DNA and protein contamination without the treatment of DNase I and Proteinase K (Fig. 1A-E). Unlike the other methods described above, no fluorescence was observed in the wells of the gels loaded with RNA extracted through the optimized protocol (Fig. 2).

The centrifugation of the LiCl-precipitated RNA dissolved in TRIzol was a crucial step in removing the DNA. The second chloroform, or CIA extraction after the extraction with TRIzol, was necessary to remove the contaminants completely. Proteinase K often separates proteins from nucleic acids and inhibits ribonucleases [44]. DNase and Proteinase K use usually demand extended treatment with phenol:chloroform: isoamyl alcohol, as has been reported in several plants [8, 43–45].

Most protocols, including the kit formats, suggested heating the samples in the extraction buffer at 65 ^o^C for up to 30 min. In our study, heat incubation for 30 min did not yield good-quality RNA from *Conocarpus erectus*. In *C. erectus*, the pre-warming of the buffer at 65 ^o^C for 5–10 min yielded good-quality of RNA (Table 2). In the case of other plant species, the pre-warming of the buffer was sufficient, and the heat incubation did not result significant change in the quality and quantity of the RNA extracted (Data not shown). *C. erectus* is reported as a source of polyphenols and reported to contain more than 12 phenolic compounds, mainly flavonoids and tannins [21, 22]. During extraction with other methods, in the case of *C. erectus*, the pellet became brown, possibly due to the biochemical changes by heat activation of some of the compounds in tissues and their binding to the nucleic acids.

During the RNA extraction, the RNase activity should be inhibited; if not, it will result in the smearing of RNA. The present protocol used 5% β-ME and 0.5 g/l spermidine in the CTAB extraction buffer. β-ME, a potent reducing agent, plays a significant role in the RNA extraction protocols that irreversibly denature RNases by reducing disulfide bonds and destroying the native conformation required for enzyme functionality and also other contaminating proteins released during tissue disruption and homogenization [40, 46, 47]. The use of 1–2% β-ME was reported as effective in most protocols [4] but relied on even the tissue types [37]. Ouyang et al. [40] documented the smearing of RNA from the flower, fruit, leaf, and bud tissues of *Neolamarckia cadamba*, even at 5% β-ME. Siles et al. [43] reported the efficacy of β-ME in avoiding the proteinase K treatment. However, in the present study, 5% β-ME was efficient in the case of *C. erectus*, and we used 5% in all other species. Spermidine plays a significant role as an RNase inhibitor [40, 46, 48]. RNA degradation depends not only on the presence of RNase but also on poly(A) length and spermidine concentration [47]. The protocol of CTAB-based RNA extraction buffer for pine needles was the first extraction buffer containing 0.5 g/l of spermidine [30]. In this case, *C. erectus* 1 g/l spermidine reduced the smearing of RNA compared to that with 0.5 g/l. Inhibition of smearing using 1 g/i of spermidine has also been reported in *Jatropha curcas* [49]. The efficacy of a higher concentration of spermidine has been reported in Maqui berry [50] and *N. cadamba* [40].

In the present study, the integrity of the extracted RNA was assessed by the visualization of ribosomal RNA bands on 1% agarose gel and Agilent 2100 Bioanalyzer microfluidic electrophoresis chip. A sharp and distinct cytosolic and plastid ribosomal bands, i.e., DNA-free, intact 28S (4.5 kb) and 18S (1.9 kb), with a double intensity of 28S rRNA band to 18S rRNA band on the agarose gel were considered as good quality RNA (Fig. 2). The extracted RNA using the optimized protocol of the present study was of good quality with the above standards (Fig. 2). In the cases of kits and TRIzol reagents extracted RNAs, the 28S and 18S rRNA bands were not intact and exhibited degradation, usually due to polysaccharides and polyphenols.

**Fig. 3:**
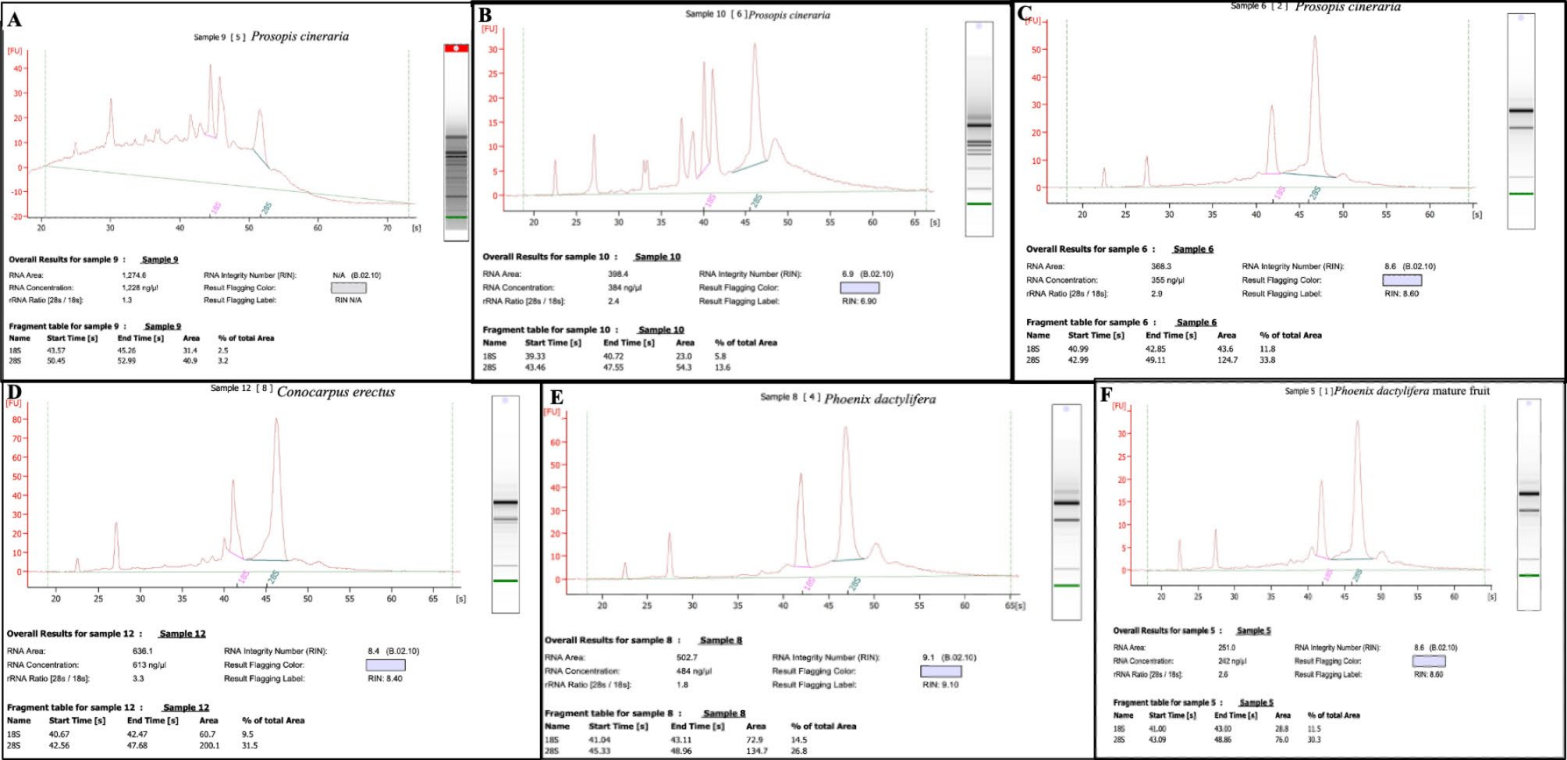
Analysis of the RNA samples from different plant species using Bioanalyzer. (A) Qiagen kit protocol; (B) CTAB method. C-F. Optimized (CTAB-TRIzol) method of the present study

The RIN values of the RNA samples of *P. cineraria* and *P. dactylifera* using the modified protocol ranged from 7.6 to 9.1, while that of *C. erectus* ranged from 7.1 to 8.6. In the present study, the results of the Nanodrop values and gel electrophoresis were corroborated with the RIN analysis of the RNA samples. RNA isolated with the optimized protocol displayed the highest peak corresponding to the 28S rRNA and 28S:18S ratio of 1.8. RIN values of 7 to 9.1 indicated the integrity of RNAs (Fig. 3C-F). RNA with a RIN value above 7.0 is required to produce good results in next-generation sequencing analysis. The RNA samples of the other protocols showed additional peaks between the 18S and 5S bands, which may be due to the degradation of 28S or 18S rRNA (Fig. 3A,B). The gel electrophoresis and the RIN analysis confirmed the high quality of RNA from all the species, which authenticated the modified protocol.

### Extraction of RNA samples with respect to the seasons from the desert-growing tree

The metabolic profile of the trees varies in the growing season, and it is one of the determinant factors of the quality of the RNA. We checked the efficacy of the optimized protocol to extract good-quality RNA from the desert tree, *P. cineraria* covering all seasons (Table 3). In the case of trees growing under stress conditions further enhance the difficulty to obtain good quality RNA due to the high metabolic profile to negate or minimize the adverse impact on cellular functions. The protocol resulted in good quality RNA from leaves > 20-year-old desert tree *P. cineraria* collected in alternate months starting from January of the year 2021, which covered the different seasons, especially the very hot and dry summer with a temperature around 50 ^o^C. The extraction of the RNA from leaf samples collected in August and October necessitated two CIA extractions following the TRIzol extraction of the optimized method.

**Table 3:** Quality and quantity of RNA extracted from leaf tissues of desert-growing *P. cineraria* collected in different months using the optimized protocol of the present study

Date of sample collection and temp (^o^C)	A_260/280_ ratio	A_260/230_ ratio	RNA yield (ng/µl)
January (22)	2.17 ± 0.08	2.25 ± 0.09^a^	1010.1 ± 31.6^b^
March (30)	2.12 ± 0.05	2.21 ± 0.06^a^	1221.5 ± 37.1^a^
May (35)	2.16 ± 0.08	2.14 ± 0.07^a^	997.6 ± 37.4^c^
July (44)	2.10 ± 0.08	2.06 ± 0.09^ab^	899.8 ± 27.6^d^
September (41)	2.09 ± 0.06	2.02 ± 0.05^b^	908.6 ± 32.9^d^
October (38)	2.09 ± 0.04	2.17 ± 0.08^a^	1000.1 ± 36.4^b^
December (24)	2.12 ± 0.07	2.14 ± 0.08^a^	991.8 ± 29.8^c^

Data represent the mean ± SE of 3 replicates each of two biological samples. RNA dissolved in 50 µl nuclease-free water. Values followed by different letters are significantly different at the 5% level

### Application of the protocol to different plant species and tissues

The RNA yield and quality rely on the type and age of tissues. The young leaves, especially the seedlings, are the prime choice for extracting intact RNA free from the impurities inhibitory to molecular applications [4]. The RNA of the present studies was extracted from mature leaves. The mature tissues, usually with high polyphenols and polysaccharides, hinder the extraction of pure and intact RNA, and it is difficult to obtain the sequence quality [4]. According to Torales et al. [19], tissue age (i.e., young freshly expanding vs. mature fully expanded but non-senescing tissue) had only a weak effect on RNA quality and no apparent effect on sequencing results in *Prosopis alba*. In their study, the younger leaves gave higher yields of RNA, and the impact of tissue age on RNA quality was observed in RIN values.

Extraction of RNA from leaves of various plant species, fruit (mature plum), tuber (*Curculigo orchioides*), rice seeds, the endosperm of coconut, and different tissues of Date palm such as root, flowers, floral parts (anther and pollen), fruits (immature and mature), and seeds were attempted with the optimized protocol (Additional file 1). The quality of the RNA using the optimized protocol was good and is useful to proceed with the downstream applications (Additional file 1). The extraction of high quality RNA from different plants and tissues, which are considered as difficult-to-extract and also from plants exposed to different seasons authenticate the protocol’s extendibility to several plants, rich in polyphenols and polysaccharides.

### Validation by downstream applications

The outcome of purified RNA on downstream applications such as real-time RT-PCR, microarray analysis, next-generation RNA sequencing (RNA-Seq), northern blotting, and cloning authenticate a protocol. The RNA extracted from stress-tolerant *P. cineraria* was used for downstream applications such as cDNA synthesis, gene cloning, cDNA library construction, and RT-qPCR and transcriptome profiling validated the modified RNA extraction protocol.

**Fig. 4:**
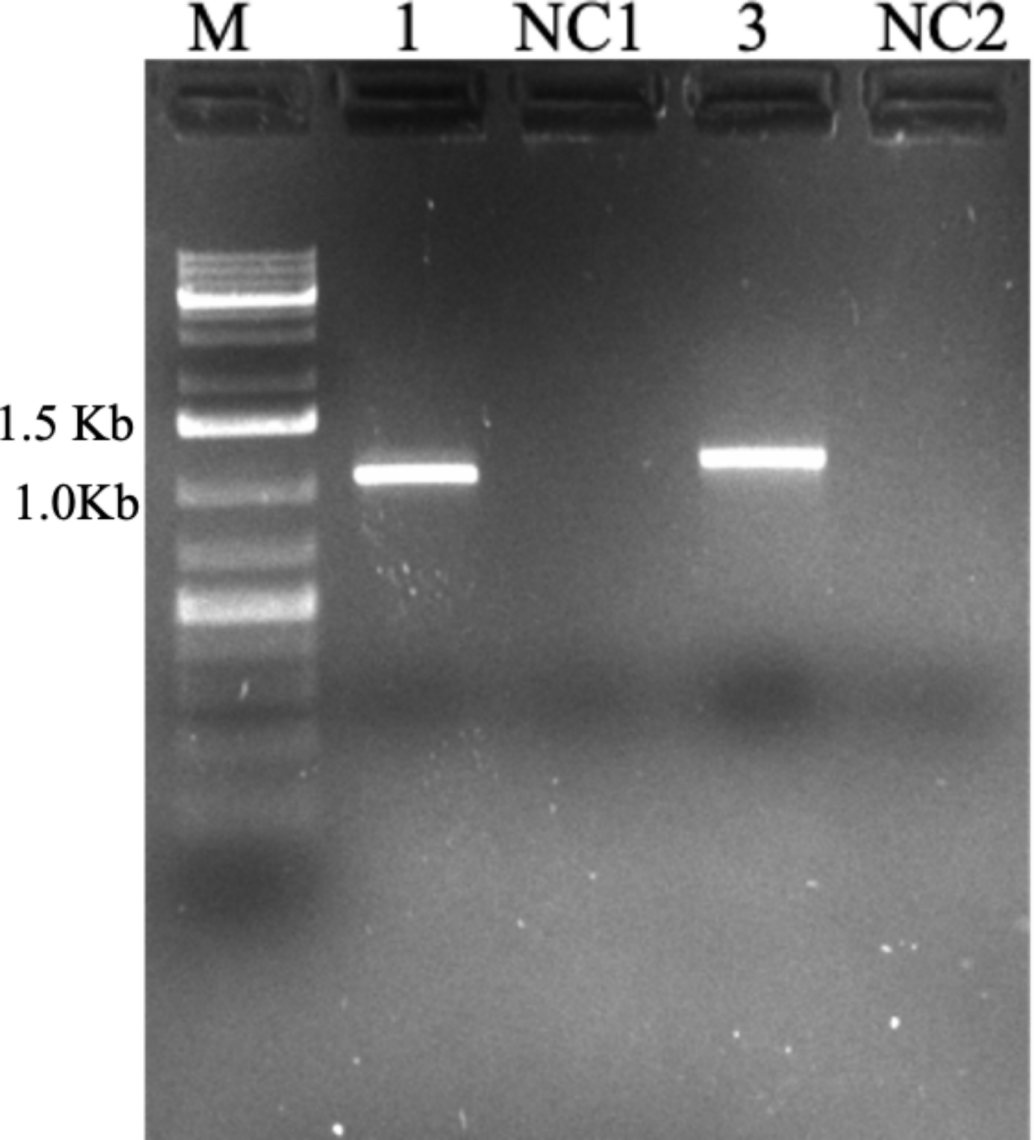
Validation of the RNA quality by amplifying *HSF6a* and *HSF7b* from the cDNA synthesized from *Prosopis cineraria* RNA using gene-specific primers (1) *HSF6a* (1028 bp); (2) *HSF7b* (1104 bp); NC1 & NC2 are negative control for *HSF6a* and *HSF7b*, respectively. M – 1 Kb Plus DNA ladder

**Fig. 5:**
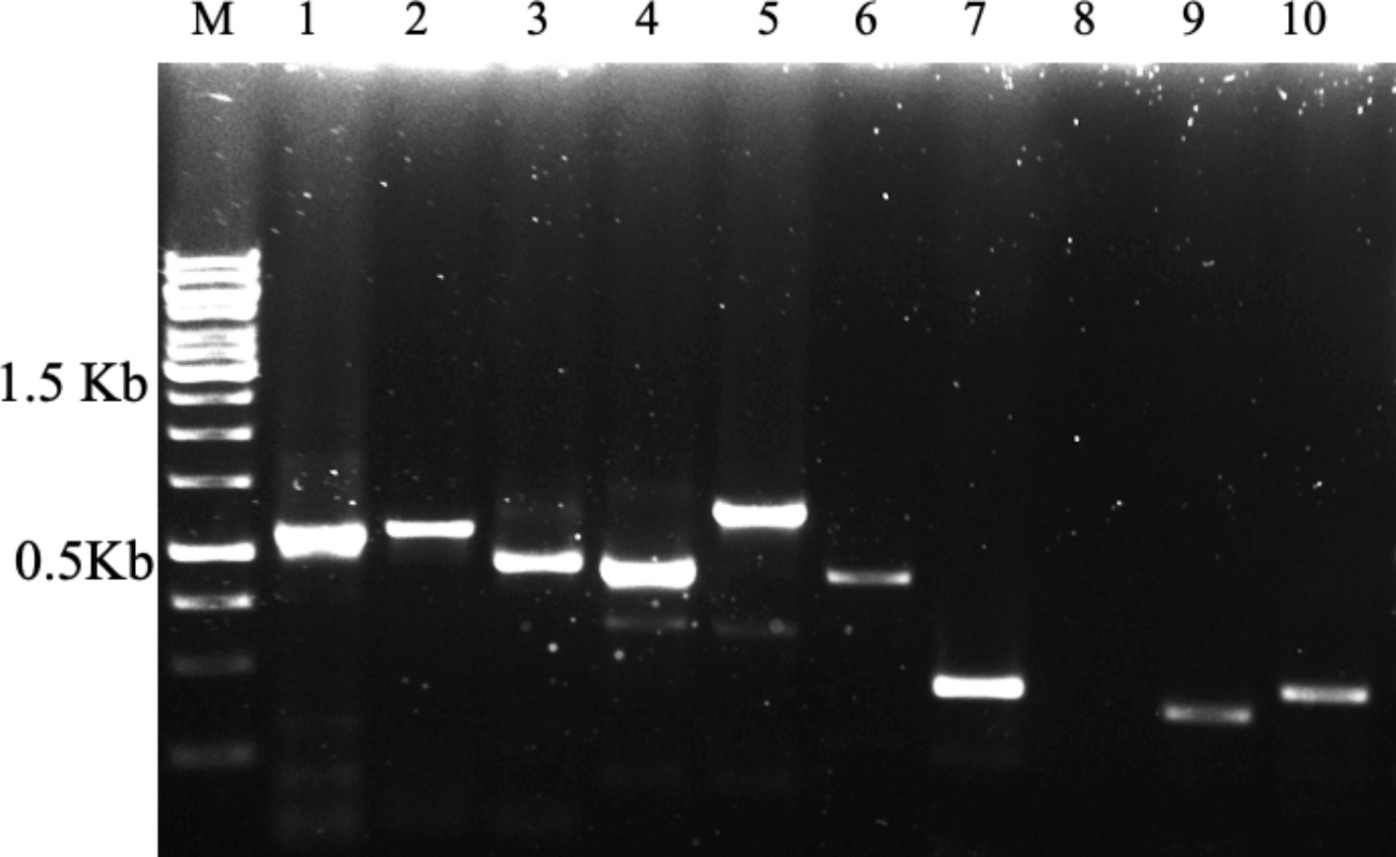
Validation of the RNA quality by the cDNA library preparation from *Prosopis cineraria* RNA. 1–10 - Amplification of the clones using M13-R and M13-R primers. M – 1 Kb Plus DNA ladder

The quality of the RNA was evaluated by synthesizing cDNA from the extracted RNA of *P. cineraria*. Heat shock factors (*HSF6a* and *HSF7b*) using gene-specific primers (Table 1) were amplified from the cDNA synthesized (Fig. 4). The gel-purified amplicons were cloned, and the sequences were confirmed by sequencing (Additional File 2) followed by a BLAST (NCBI) similarity search. The cDNA library was constructed using the *P. cineraria* RNA, and the clones confirmed by PCR using the M13F and M13R (Fig. 5) were sequenced, and several genes imparting abiotic tolerance were identified and cloned for crop modification (unpublished).

**Fig. 6:**
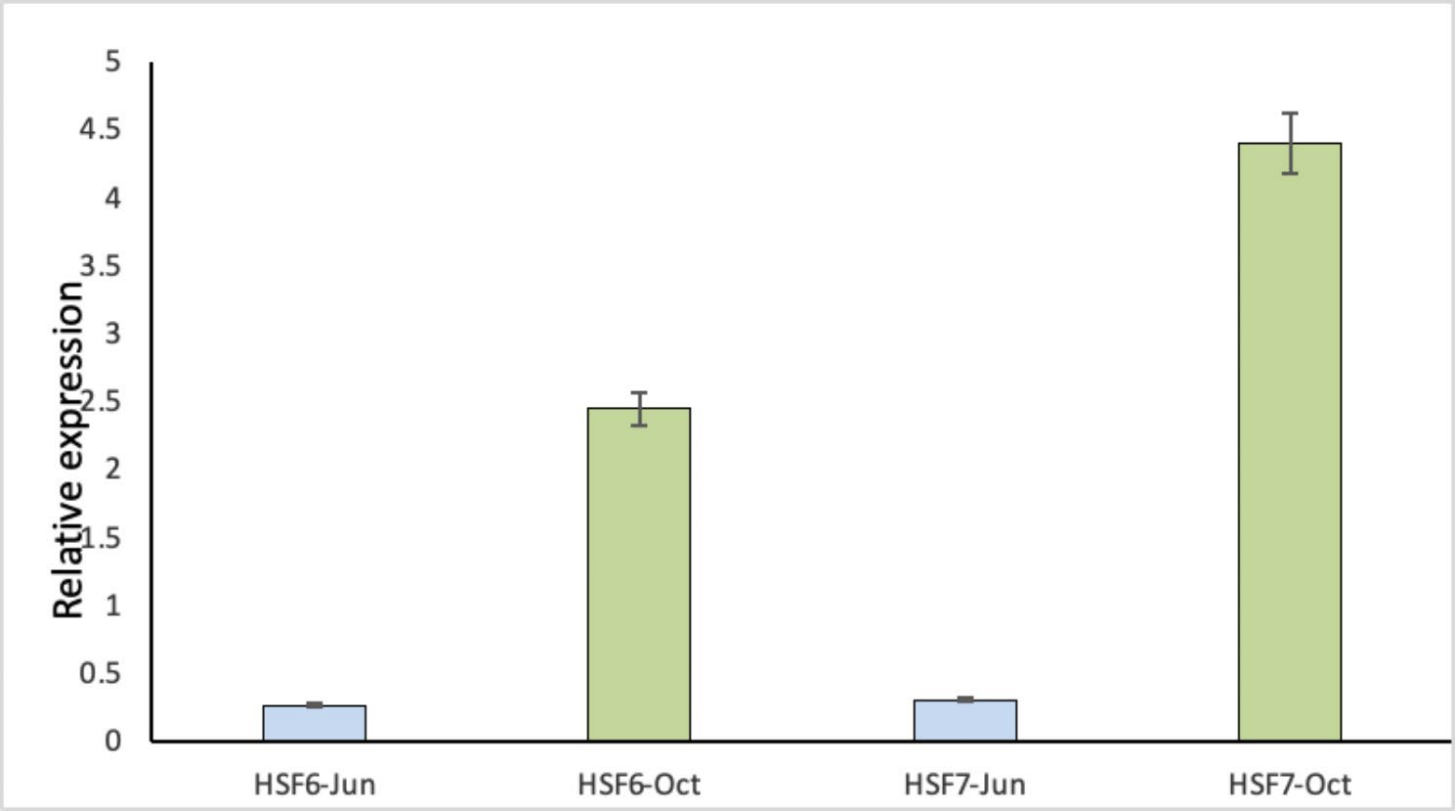
Expression of *HSF6a* and *HSF7b* of the RNA samples collected in June and October 2021 samples of *Prosopis cineraria* RNA. Data represent the mean of 3 biological replicates. Actin was used as the reference gene

High-quality, intact RNA in sufficient amounts is the key for gene expression studies to understand the biological processes, especially the upregulation and downregulation of genes with respect to specific tissues and also under environmental stimuli. The cDNA synthesized using the QuantiTect Transcription kit followed by Real-Time qPCR used for the expression analysis of the heat shock factors (*HSF6a* and *HSF7b*) with Actin as reference gene showed a significant difference in expression level (Fig. 6), and this validated the modified RNA extraction protocol of the present study.

The samples of RNA extracted through the modified protocol from *P. cineraria*, a total of 54 samples of three desert trees (> 20-years-old) collected in every alternate month (triplicates) from January through December of the year 2021 (Table 3) qualified the sequence quality parameters (Novogene) for different NGS platforms, and the analysis is in progress (Data not shown).

## Conclusion

No universal protocol is available for isolating total RNA as the plants vary significantly in their structural and biochemical profile. However, the modified protocol yielded high-quality DNA-free intact RNA from various plant species, tissue types, and with respect to season, which was authenticated by bioanalyzer results and successful downstream use. The protocol is useful for extracting DNA-free intact RNA from plants with high phenolics, starch, and polysaccharides.

### Electronic supplementary material

Below is the link to the electronic supplementary material.

Additonal file 1: RNA extracted from different plant species/tissues

Additional file 2: The sequences of HSFs

## Data Availability

Available on request to the corresponding authors.
