# Supplementary material for: DNA-free high-quality RNA extraction from 39 difficult-to-extract plant species (representing seasonal tissues and tissue types) of 32 families, and its validation for downstream molecular applications
Source: Plant Methods. 2023 Aug 11;19:84. doi: 10.1186/s13007-023-01063-5 (PMC10416385; doi:10.1186/s13007-023-01063-5)
Supplement: Supplementary file 1 — Additonal file 1: RNA extracted from different plant species/tissues [file 13007_2023_1063_MOESM1_ESM.docx]

**Additional File 1** Quality and quantity of RNA extracted from different plant species using the optimized protocol

| Plant Species | Family | Tissue type | A_260_/_280_ ratio | A_260/230_ ratio | DNA yield (ng/μl) |
| --- | --- | --- | --- | --- | --- |
| *Phoenix* *dactylifera* | Arecaceae* | Male flower  Female flower | 2.11±0.06 2.06±0.05 | 2.19±0.09/ 2.09±0.07 | 1098.6±28.5 891.1±36.1 |
|  |  | Anther | 2.11±0.06 | 2.11±0.09 | 654.8±22.4 |
|  |  | Pollen | 2.11±0.06 | 2.05±0.03 | 468.5±29.1 |
|  |  | Immature fruit  Mature fruit | 2.13±0.06  2.12±0.04 | 2.06±0.06 2.08±0.05 | 1100.6±36.2 974.1±27.7 |
|  |  | Seed | 2.01±0.07 | 1.95±0.06 | 320.2±11.9 |
|  |  | Root | 2.00±0.05 | 2.01±0.06 | 391.6±24.2 |
| *Curculigo orchioides^#^* | Hypoxidaceae* | Tuberous roots | 1.99±0.08 | 1.99±0.09 | 311.7±14.6 |
| *Orzya sativum* var. *indica^#^* | Poaceae* | Seed | 1.98±0.05 | 1.94±0.06 | 308.6±19.4 |
| *Pandanus amaryillifolius^#^* | Pandanaceae* | Leaf | 2.15±0.09 | 2.21±0.07 | 769.6±29.8 |
| *Musa paradisiaca^#^* | Musaceae* | Leaf | 2.01±0.05 | 2.11±0.09 | 801.6±32.4 |
| *Vanilla planifolia^#^* | Orchidaceae* | Leaf | 2.10±0.08 | 2.08±0.06 | 619.4±21.8 |
| *Cocos nucifera^$^* | Arecaceae* | Endosperm | 1.97±0.06 | 1.93±0.05 | 594.1±17.4 |
| *Elaeis guineensis^#^* | Arecaceae* | Leaf | 1.81±0.05 | 2.09±0.08 | 491.6±31.6 |
| *Memecylon umbellatum^#^* | Melastomaceae | Leaf | 2.11±0.08 | 2.11±0.06 | 796.0±27.7 |
| *Prunus salicina^$^* | Rosaceae | Mature fruit | 2.00±0.07 | 1.95±0.04 | 692.6±16.4 |
| *Solanum lycopersicum^#^* | Solanaceae | Immature fruit  Mature fruit | 2.11±0.09 2.04±0.06 | 2.12±0.04 2.01±0.06 | 1009.6±31.2 971.3±28.9 |
| *Prosopis juliflora* | Fabaceae | Leaf | 2.15±0.05 | 2.23±0.08 | 1010.3±33.6 |
| *Azadirachta indica* | Meliaceae | Leaf | 2.16±0.07 | 2.19±0.08 | 684.2±21.7 |
| *Gossypium hirsutum* | Malvaceae | Leaf | 2.11±0.07 | 2.14±0.09 | 692.1±28.8 |
| *Ziziphus jujuba* | Rhamnaceae | Leaf | 2.19±0.10 | 2.31±0.11 | 736.4±31.6 |
| *Haloxylon persicum* | Amaranthaceae | Leaf | 2.13±0.07 | 2.21±0.08 | 729.1±19.4 |
| *Leptadenia pyrotechnica* | Ascleipidaceae | Immature stem | 2.09±0.06 | 2.11±0.09 | 699.3±22.2 |
| *Calotropis procera* | Ascleipidaceae | Leaf | 2.16±0.06 | 2.17±0.07 | 901.7±29.6 |
| *Thevetia peruviana* | Apocynaceae | Leaf | 2.12±0.09 | 2.20±0.06 | 824.3±21.6 |
| *Tamarix aphylla* | Tamaricaeae | Leaf | 2.10±0.05 | 2.14±0.07 | 691.1±23.0 |
| *Terminalia catapa* | Combretaceae | Leaf | 2.17±0.09 | 2.00±0.07 | 648.8±19.9 |
| *Caesalpinia pulcherrima* | Caesalpiniaceae | Leaf | 2.14±0.05 | 2.07±0.08 | 712.17±24.0 |
| *Delonix regia* | Caesalpiniaceae | Leaf | 2.12±0.05 | 2.29±0.10 | 882.6±26.6 |
| *Piper nigrum* | Piperaceae | Leaf | 1.98±0.07 | 1.94±0.04 | 622.4±18.4 |
| *Cassia fistula* | Caesalpinaceae | Leaf | 2.08±0.08 | 2.15±0.06 | 725.7±18.7 |
| *Mangifera indica* | Anacardiaceae | Leaf | 2.01±0.06 | 2.00±0.08 | 686.8±20.7 |
| *Acacia nilotica* | Mimosae | Leaf | 2.10±0.08 | 2.02±0.08 | 619.7±25.8 |
| *Eucalyptus* sp. | Myrtaceae | Leaf | 2.01±0.08 | 2.00±0.06 | 666.1±26.7 |
| *Salicornia bigelovii* | Salicorniaceae | Leaf | 2.11±0.10 | 2.21±0.09 | 991.9±32.7 |
| *Moringa peregrina* | Moringaceae | Leaf | 2.16±0.08 | 2.07±0.05 | 798.6±29.8 |
| *Ficus religiosa* | Moraceae | Leaf | 2.10±0.07 | 2.19±0.06 | 716.3±30.5 |
| *Zygophyllum coccineum* | Zygophyllaceae | Leaf | 2.09±0.07 | 2.08±0.09 | 798.1±27.8 |
| *Avicennia marina^#^* | Acant­haceae | Leaf | 2.07±0.09 | 2.02±0.07 | 751.6±21.4 |
| *Annona squamosa^#^* | Annonaceae | Leaf | 2.10±0.09 | 2.06±0.09 | 699.8±20.0 |
| *Sesuvium verrucosum^#^* | Aizoaceae | Leaf | 2.11±0.04 | 2.09±0.07 | 786.1±30.1 |
| *Litchi sinensis^#^* | Sapindaceae | Leaf | 2.09±0.06 | 2.08±0.07 | 697.9±28.2 |
| *Tecoma stans* | Bignoniacae | Leaf | 2.16±0.08 | 2.21±0.09 | 871.2±27.0 |

*Monocots. All others are dicots. *^$^*Collected from the fruit shop. ^#^Tissues from plants grown in the greenhouse. All other tissues were collected from plants grown around the Center and near places in Al Ain, UAE. Data represent the mean of 3 replicates. Values are Mean±SE. Extracted RNA dissolved in 50 μl nuclease-free water.
