# Supplementary material for: DNA-free high-quality RNA extraction from 39 difficult-to-extract plant species (representing seasonal tissues and tissue types) of 32 families, and its validation for downstream molecular applications
Source: Plant Methods. 2023 Aug 11;19:84. doi: 10.1186/s13007-023-01063-5 (PMC10416385; doi:10.1186/s13007-023-01063-5)
Supplement: Supplementary file 2 — Additional file 2: The sequences of HSFs [file 13007_2023_1063_MOESM2_ESM.docx]

**Additional File 2**

Sequences of *HSF6a* (1023 bp) and *HSF7b* (1104 bp) amplified from the cDNA synthesized from *Prosopis* *cineraria* RNA using gene-specific primers

> *PcHSF6a* (1023 bp)

ATGAATCGTATCGATGATGGTCCTGTGATTGTGAAGGAGGAGTTTCCTGGTGAAGGGTTGGTTGCTCCACAGCCGCTTGAGGGGCTTCATGAAAGTGGTCCTCCACCTTTTCTCACAAAGACTTATGACATAGTTGATGATGTTTCCACTGATGAGATAGTTTCATGGAGCAGAGGAAACAACAGCTTCGTTGTCTGGGATCCTCAAGCTTTTTCCATCTCTCTTCTCCCCAAATACTTCAAACACAACAACTTCTCCAGCTTTGTCAGACAGCTCAACACCTATGGGTTCAGAAAGGTGGATCCTGACAAATGGGAGTTTGCTAATGAAGGTTTTCTTAGAGGACAGAAACATCTATTGAAGATCATAAGGAGGAGGAAGAGCAGTCATCAACCTCAGGCAGTACAACAATCTATGGAACATTGTGTTGAAGTGGGGAGGTTTGGATTAGATGGAGAAATCGATGGATTGAGACGTGACAAGCAAGTTCTGATGATGGAGCTGGTGAAACTTAGACAGCAACAGCAGAACACAAAAAACCACCTTCAATCAATGGAAAGTAGGCTCAAAAAGACTGAACAGAAACAGCAGCAGATGATGAAGTTCTTGGCAAAGGCAATGCAGAACCCCAACTTCTTGCAACAATTGGTTCAAGAAAGGGAGTGGAGGAAGGAGCTGGAGGAAGCTATTTCCAGCAAGAGAAGAAGGCCTATTGATGAAGGGCCTTGTGATGCTGAAGAATTTTCAGCTTTGGTTAAACTTGAACAGGAGGAGTGCAATGAGATTTCAGAGCTTGAGGTTTCTGACATAGAGCTTGCTATGAACATGGAGGAACAAAGTGGAAGCCAAGAGAACACTTATGGTGAAGAAGAGAAAGGAGGCGAAGATGAAAGTAGAAATGAAGGCATTGAAGAAGTACTGTGGGAAGAACTTCTGAATGAGGGTGTTGAAGAAGATCTACTAGCATTGGAAAATGATGATAATGAACATGTAACTTTGTTGTCTGAAGAACTTGGTTATTGA

>*PcHSF7b* (1104 bp)

ATGAACTATTTGTACCCTGCGAAAGAAGAGTACTTTGAATCGCCACCACCATCCTCCTCATCGTCGCCAACGTCTCCCGGTGAGTCAGCAATGGTTTTTCTTCCGAGGACAATGGAGGGGCTTCACGAAATAGGGCCACCTCCATTTCTGACCAAGACCTATGATGCCGTGGAGGACCCTACCACTAGCCACATAGTGTCATGGAGCAGAGGTGGCGCCAGCTTTGTGGTTTGGGATCCTCACGCTTTCTCCAGAGACCTTCTCCCTCGATACTTCAAGCATATCAATTTCTCCAACTTTGTAAGGCAACTAAACACTTACTGCTTTAGAAAGATTGATCCCGATAGGTGGGAGTTTGCATACGAAGGATTCCTGAGAGGACACAGGCACCTTTTGGCAAACATCAGGAGAAGAAAGCAACCTTCTCAGGCTTCCACTTCTCAACAAGGACAAGGGCACTGTGTTGAACTTGCTCGTTGTGGACTTGATGAAGACGCTGATCGTTTAAGGTGTGACGAGCAGGTGCTGATGATAGACCTTGTGAAGCTGAGACAGCAGCATCAGAATACGAGGTCGTACCTTGAGGAAATGGAGGAGAGGCTACGAGGGACAGAAATTAAGCAGCAGCAGATGATGTCTTTCTTGGCTAGAGCTTTGAAGAACCCCACTTTCATCCAGCAGCTACTCCAGCAGAAAGAGAAGAGGAAGGAGCTTGAAGAGGCAATGTCGAAGAAGAGGAGAAGACCAATCGAGCAAGGAGCAAGTGGGGTTGGAGAATCAAGCATTGGAAGGGAAGGAAGAAACAGCGTTAAAGTGGAGTGCCAAGTGTTTGGGGAATATGGATTTGGAGTCTCAGAGTTGGAAGTGTTGGCTTTGGAGATGCAAGGTTATGGTAGGGGAAAGAGGGAACAAGAGGAAGAGCCTGAGGCACTAGAATCACAGGAGAGGTTGGAGAAAGAACTTGATGAAGGTTTCTGGGAAGAACTGTTCAGTGAGGGTTTTGAGGGTGAATTAGATATTCCCACTTCACAAGACCCAGATGAAGAAGAAGATGTCAATGTGTTAGCCAATCGCTTTGGTTACTTAGGGTCAAGTCCTAAATGA
